# Prevalence and Burden of Primary Headache Disorders in Kuwaiti Children and Adolescents: A Community Based Study

**DOI:** 10.3389/fneur.2019.00793

**Published:** 2019-07-30

**Authors:** Jasem Yousef Al-Hashel, Samar Farouk Ahmed, Raed Alroughani

**Affiliations:** ^1^Neurology Department, Ibn Sina Hospital, Kuwait City, Kuwait; ^2^Department of Medicine, Kuwait University, Kuwait City, Kuwait; ^3^Division of Neurology, Amiri Hospital, Kuwait City, Kuwait; ^4^Department of Neuropsychiatry, Minia University, Minia, Egypt

**Keywords:** primary headache, children, adolescent, prevalence, migraine, tension type headache

## Abstract

**Background/Objective:** Primary headaches are common in the pediatric and adolescent population and can be disabling for them and their families. We aimed to assess the prevalence and burden of primary headache disorders among children and adolescents in Kuwait.

**Methods:** A cross-sectional community-based study included Kuwaiti population aged 6–17 years. They were randomly recruited from all six governorates of Kuwait using stratified multistage cluster sampling. The Headache-Attributed Restriction, Disability, and Social Handicap and Impaired Participation (HARDSHIP) questionnaire for children and adolescents was used to collect the data.

**Results:** Data were collected from 3,423 subjects; 664 subjects were diagnosed as having primary headache disorders. The mean age was 12.61 ± 2.51 years and 64.2% were females. One year prevalence of headache was 19.4%. It was significantly prevalent in females compared to males (25.2% vs. 13.8%; *P* < 0.001). Primary headache disorder significantly increased in age group 12–17 when compared to age group 6–11 years (25.8% vs. 10.4 %; *p* < 0.001). One year primary headache prevalence showed non-significant differences in both males and females in age group 6–11 years (10.1% in males vs. 10.6% in females; *P* < 0.79), while it was significantly higher in female vs. males (38.1% vs. 15.8%; *P* < 0.001) in age group 12–17 years. Migraine prevalence was 10.9% followed by tension type headache (TTH) 6.2% and chronic headache 0.9%. Medical care utilization was reported in 67% of our cohort. The majority (95%) of the patients received symptomatic drugs for headache attacks and only 7.5% used preventive medication. The students with headache lost a mean of 1.29 ± 1.23 days of school, reported mean of 1.16 ± 1.50 days they could not do activities they had wanted to. Their parents lost a mean of 1.01 ± 1.02 days of work because of headaches of their children during the preceding 4 weeks of the study.

**Conclusions:** The estimated 1 year prevalence of headache was 19.4% overall. Primary headache prevalence increased with age and it was more prevalent in female adolescents compared to males of the same age. Headache disorders in children/adolescents affect school and social activities as well as their parents work. The awareness for early diagnosis and preventive medications for headache in this age group may reduce the headache burden.

## Introduction

Pediatric headaches are common and may cause significant distress and disability in children and adolescents and their families (1). Prevalence of headache increases throughout childhood with a peak at 11–13 years old in both sexes (2). Both migraine and tension type headache (TTH) are the most predominant headaches (3). It was reported that 6.1 to 13.6% of children have migraine and 9.8 to 24.7% have TTH (4). Migraine is more disabling and required more medication use than TTH (5). Ratio of girls to boys in all headaches is 1.5:1 while it is 1.7:1 in migraines (6). The male to female ratio is 1:3 for migraine and 4:5 for TTH after 12 years of age (7, 8). Headache disorders in children and adolescents are associated with economic burden to the families and the society (9). Pediatric headache has a bad adverse impact on the children and their families (10). Primary headache disorders also influence negatively school and social functions and have a negative effect on quality of life. We have little information on pediatric headache compared to our knowledge of headache epidemiology in adults. Accurate estimate of the pediatric headache prevalence in Kuwait will help estimating the magnitude of the problem, improving diagnosis and treatment, and reducing its economic and social burden. We aimed to assess the prevalence and burden of primary headache disorders in Kuwaiti children and adolescents aged 6 to 17 years.

## Methods

A cross-sectional population-based door-to-door survey was conducted. Multistage random cluster sampling was used. Population of the study included Kuwaiti children and adolescents aged 6–17 years and living in Kuwait for the last 6 months.

The state of Kuwait consists of 6 governorates. Each governorate is divided into areas and each area has several streets. We collected samples from all the 6 governmental divisions in Kuwait. A random selection of 3 areas within all 6 governorates was performed. The main street was selected in each area, and then one direction was selected to proceed. At the last stage, we choose the first house randomly. It was selected by using the “spin the bottle” method. After that each house was selected along the line in the same direction until the projected sample size was achieved. Research team visited the houses unannounced at different times. The head of the family in each visited house was asked to list all the family members within the age range 6–17 years. One of each list was randomly invited to participate by the sealed envelopes method. The sealed envelope contained the eligible list, from which the head of the household picks one randomly.

To collect study data, trained interviewers conducted face-to face interviews using Child and Adolescent HARDSHIP questionnaires (11).

The Child HARDSHIP for children aged 6–11 years and Adolescent HARDSHIP questionnaire for adolescents aged 12–17 years were used. These questionnaires were used to evaluate the prevalence of headache, as a diagnostic tool to differentiate among types of headache and their impact on quality of life (11). To screen for headache, we asked about the duration and 1 year prevalence. To diagnose headache type according to ICHD-II criteria (12), we asked questions about headache frequency and duration, headache characteristics, associated symptoms and use of acute medication.

To assess the diagnostic accuracy and to validate the questionnaire in our community, face-to face interviews in random subsamples of participants with migraine or TTH according to questionnaire diagnoses was performed. Fifty subjects aged 6–11 years and 50 subjects aged 12–17 years were interviewed by experienced headache specialists Jasem Yousef Al-Hashel.

**Figure 1:**
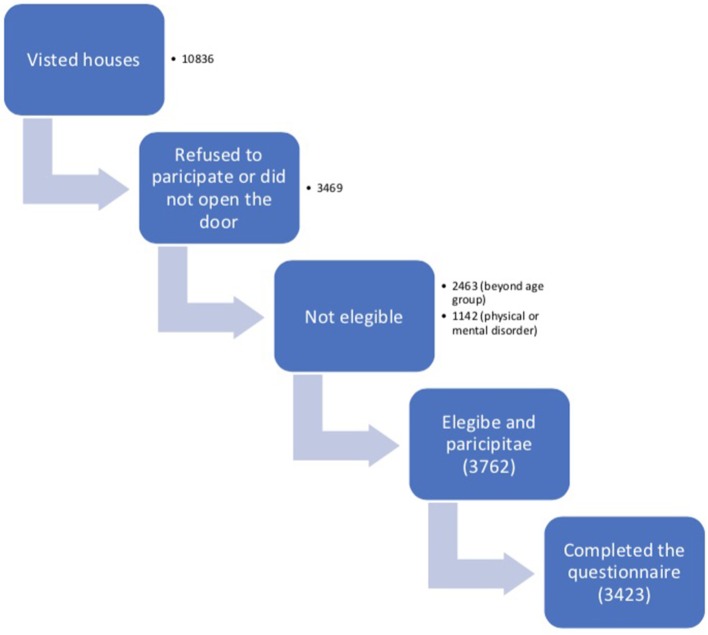
Flowchart of participation.

The sample comprised 3,423 subjects (0.64% of the pediatric population). Our interview team visited 10,836 households during 4 months, of which 3,469 (32.1%) refused to participate or did not open the door. Out of the 7,367 remaining, 3,605 (48.9%) were not eligible either because of their age (2,463) or because of their mental or physical disorders (1,142) that may be a cause of headache. Of 3,762 eligible households identified, 3,423 (90.9%) completed the questionnaire. Out of 7,367 visited houses only 3,423 completed the questionnaire with a participation rate of 46.5%. Flow chart in Figure 1.

The faculty of Medicine, Kuwait University approved the study. Eligible participants and their parents received a simple explanation about the aim of the study being considered an ethical issue. Written informed consent was obtained from all participants and their parents before we asked the survey questions.

## Statistical Analysis

According to the Kuwait Bureau of Statistics 2015 report, the total population of Kuwaiti aged 6–17 years in 2014 was 535,692. Boys represented 272,610 (50.89%) of the population and girls are 263,082 (49.11%). We estimated a total sample size of 3,000 using a special formula based on reported prevalence of headache from previous international epidemiological studies, which is around 54.4% and of migraine, 9.1% (13). Then, the sample was increased by 10% to overcome the problem of non-response and missing data.

Completed questionnaire data were entered into SPSS version 20.0. Double check of all data was made, with inconsistencies reconciled by reference to the original documents. Error rate of 1.8 % was identified. One year prevalence for primary headache disorders as percentages with 95% CIs. Adjusted prevalence for gender and age according to the participation of the pediatric Kuwaiti population was reported. Age in years was analyzed as a categorical variable (6–11 and 12–17). Headache frequency was recorded as continuous data in days affected per month. We recorded the duration of headache as continuous data in hours. We recorded headache frequency in days over the preceding 3 months, and typical headache intensity on a verbal rating scale (“not bad,” “quite bad,” and “very bad”). We used proportions, 95% CIs, means, and SDs to summarize the distributions of variables and chi-square, for significance of differences. *P* < 0.05 was considered as statistically significant.

## Results

The mean age of total sample (3,423) was 12.48 ± 3.03. Males represented 1,734 (50.7%) and girls represented 1,689 (49.3%) of the sample. A total of 664 subjects was diagnosed with primary headache disorders with mean age 12.61 ± 2.51 years, of which 35.8% were males (238) and 64.2% were females (426).

Table 1 shows that 1 year prevalence of headache was 19.4% in the headache sample. It was significantly prevalent in females compared to males (*P* < 0.001). Headache significantly increased in the age group 12–17 when compared to 6–11 years (*p* < 0.001). The 1 year prevalence of primary headache showed non-significant difference in both males and females in the age group 6–11 years (*P* < 0.79), but it was significantly higher in females compared to males (*p* < 0.001) in the age group 12–17 years.

**Table 1:** One year prevalence of primary headache disorders stratified by age and gender.

**Primary headache disorder**	**Total**			**Male**			**Female**			***P***
**Age (years)**	**Participant populations**	**Headache cases**	**Prevalence % (CIs 95%)**	**Participant populations**	**Headache cases**	**Prevalence % (CIs 95%)**	**Participant populations**	**Headache cases**	**Prevalence % (CIs 95%)**	
6–11	1,411	146	10.4% (8.9.5–12.1)	616	62	10.1% (7.9–12.7)	795	84	10.6 (8.6.5–12.9)	0.79
12–17	2,012	518	25.8% (23.9–27.0)	1,118	177	15.8% (13.8–18.1)	894	341	38.1% (35.02–41.4)	0.001^*^
Total	3,423	664	19.40 (18.1–20.8)	1,734	239	13.8 (12.2–15.5)	1,689	425	25.22 (23.2–27.3)	0.001^*^

*CIs, confidence intervals*;

* significant P-value.

Table 2 shows that migraine was the most prevalent headache, followed by TTH and chronic headache. Prevalence of migraine and TTH increased significantly at age group 12–17 when compared to age group 6–11 years (*P* < 0.001) and (*P* < 0.05), respectively. Prevalence of migraine was significantly higher among girls compared to boys (*P* < 0.001). Prevalence of TTH was non-significantly lower in males compared to females (*P* < 0.1).

**Table 2:** Observed 1 year prevalence of headache types stratified by age and gender.

**Variables**	**Primary headache** **(%) [CI 95%]**	**Migraine** **(%) [CI 95%]**	**TTH (%)** **[CI 95%]**	**Chronic headache** **(%) [CI 95%]**	**pMOH (%)** **[CI 95%]**	**Unclassified headache** **(%) [CI 95%]**	***P***
**Age group:**							
6–11 years: 1411 12–17 years: 2012	146 10.4% [8.9.5–12.1] 518 25.8% [23.9–27.0]	100 7.1% [5.9–8.6] 274 13.6% [12.2–15.2]	43 3.1% [2.3–4.1] 170 8.5% [7.3–9.8]	0 33 1.6% [1.1–2.3]	0 26 1.3% [0.9–1.9]	3 0.2% [0.04–0.7] 15 0.7% [0.4-1.2]	0.001^*^ 0.001^*^
**Gender:**							
Male: 1734 Female: 1689	239 13.8% [122–15.5] 425 25.2% [23.2–27.3]	119 6.9% [5.8–8.2] 255 1 5.1% [13.5–16.9]	96 5.5% [4.6–6.7] 117 6.9% [5.8–82.2]	12 0.7% [0.4–1.2] 21 1.2% [0.8–1.9]	8 0.5% [0.2-0.9] 18 1.1% [0.7–1.7]	4 0.2% [0.07–0. 6] 14 0.8% [0.5–1.4]	0.016^*^ 0.0001^*^
**Total:** 3423	664 19.400% [18.1–20.8]	374 10.9% [9.9–12.02]	213 6.2% [5.5–7.1]	33 0.9% [0.7–1.3]	26 0.8% [0.5–1.1]	18 0.5% [0.3–0.8]	0.001^*^

CI, confidence interval; TTH, tension-type headache; pMOH, probable medication-overuse headache;

* significant P-value.

Characteristics and burdens of primary headache were displayed in Table 3. Students with headache lost a mean of 1.29 ± 1.23 days of school, with a reported mean of 1.16 ± 1.50 days when they could not do the activities they wanted to do. Their parents lost a mean of 1.01 ± 1.02 days of work because of their children headache during the 4 weeks preceding the study.

**Table 3:** Characters and burden of primary headache in pediatric patients with primary headache disorders (no = 664).

**Variables**	**Total headache sample *N* = 664 M ± SD/No (%)**
**Gender:**	
Male Female	238 (35.8) 426 (64.2)
**Mean age in years**	12.61 ± 2.51
**Range**	6–17
**Age group:**	
6–11 years 12–17 years	348 (52.4) 316 (47.6)
Number of headache days **in the last 4 weeks**	6.51 ± 2.28
**Duration of attacks in hours in the last 4 weeks**	9.56 ± 6.56
**Number of analgesic** days **in the last 4 weeks**	6.22 ± 2.69
**Severity of headache**	
Not bad Quite bad Very bad	34 (5.1) 454(68.4) 176(26.5)
**Treatments taken**	
Symptomatic treatment Prophylactic treatment	630 (94.9) 50 (7.5)
**Use health service**	446 (67.2)
**Consultations**	
General practitioner Neurologist Arabic medicine ENT Ophthalmology More than one specialties	262 (39.5) 36 (5.4) 60 (9) 43 (6.5) 30 (4.5) 15 (2.3)
**Impact of primary headache over last 4 weeks before the survey**	
Mean lost school days Mean days of leave school early Mean days could you not do things because of headache Mean lost days of parents work because of headache	1.29 ± 1.23 1.16 ± 0.71 1.16 ± 1.50 1.01 ± 1.02

*M, mean; SD, standard deviation*.

## Discussion

Our previous epidemiological studies demonstrated that the prevalence of primary headache disorder in Kuwait is 61% in the age group 18–65 years (14). Our earlier study demonstrated the prevalence of migraine among medical students 27.9% (15). These previous results were comparable to international figures. We decided in this work to look at the prevalence of primary headache disorders in pediatric Kuwaiti population.

Our population study included 3,424 subjects, of which 1,734 were males and 1,689 were females with a mean age of 12.48 ± 3.03. Primary headache disorders in the year previous to the survey was diagnosed in 664 of them.

We reported 1 year prevalence of primary headache to be 19.4% in the age range 6–17 years. It was 10.4% in 6–11 years old and 28.8% in 12–17 years old. Our results are comparable with previously published results on the primary headache prevalence in pediatric patients in the world (7, 16–21). Pediatric headaches prevalence is widely variable, based on the methodology, genetic difference and used diagnostic criteria. The prevalence of primary headaches was 9.68%, in the age range 9–15 in China (16), 24.4% in the age range 7–15 in Sweden (7), 24% in Jordan (17), 25.5% in school children aged 7–14 years old in India (18), 29.1% among school children in Korea (19), 66% in India (20), 46.2% in children aged 6–10 in Turkey (21). This variation can be attributed to several different cultural, genetic, environmental factors, ethnological, cultural, and geographical components or methodological differences or applied diagnostic criteria. Methodological dissimilarities in the number of enrolled headache patients, the patient selection criteria, the different age ranges, and the sources of information used (parents or child) and symptom collections probably accounted for differences in the results.

Our results reported that migraine was the most prevalent (10.9%), followed by TTH (6.2%) and chronic headache (0.9%). Similarly to our study, a population study consisting of 2,466 children, aged between 3 and 18 years and which included 60% females and 40% males, showed that migraine was the most prevalent headache followed by TTH, and then other primary headaches (22). Our results are also in line with the Indian study that showed that migraine was most prevalent in their cohort followed by TTH (18). In different countries, some results reported a higher prevalence of migraines than other types of primary headaches and other studies found a higher prevalence of TTH compared to migraine (23). Our results were in line with a Chinese study (16) that showed that migraines were more common than any other headache subtypes. The 1 year prevalence of migraine in our result is 10.9%, which is comparable to previous studies. A previous review included 64 cross sectional studies with a total of 227,249 participants conducted in 32 countries and reported an overall mean prevalence of migraine of 9% (13) which is similar to our 10.9% results.

Previous studies of headaches in children showed migraine to be the most common cause of primary headache (24). Migraine prevalence was reported as 3% in Hong Kong (25), 2.9% in Jordan (17), 7.8% in India (26), and 17% in the USA (27).

In our population-based study, headache prevalence increased with age. Our survey found significant difference between the age groups (*p* < 0.01) which is in line with previous studies (28). Similarly, in a German study, the prevalence of headache was increasing with age from 39% for 7 years old to 63% for 14 years old (29). Although an increase in headache prevalence with age has been widely accepted (30), discrepancies have also been reported. Heinrich et al. (31) found no increases in the prevalence of migraines or TTH with age.

The increased prevalence of headache with age could be explained by the increasing prevalence of primary headache among girls after puberty and the prevalence of headache is more prevalent in females compared to males.

Our results showed 1 year prevalence of primary headache was significantly higher in females 25.2% compared to males 13.8%. Most of the previous studies found a higher prevalence of headache in girls (3, 18), whereas fewer studies (29, 32) did not. Our results demonstrated that girls in the age group 12–17 years had higher frequency of primary headaches vs. boys. However, no significant difference was found in the age group 6–11 years. This result showed that female adolescents are at higher risk to develop primary headache compared to female children which is in agreement with previous results (33). This increase in the female group is likely due to secondary hormonal change that take place around this age. Hormonal effect triggers migraine in female adolescents (34). Headaches are more common in adult women (35), while in children and adolescents it is debatable whether headache prevalence differs between genders. Earlier studies (36), showed that primary headaches are more prevalent in females compared to males during puberty.

Prevalence of primary headache among gender is different in different epidemiological studies. Laurell reported a higher rate TTH and migraine in females (7). However, Lyngberg, in a large epidemiologic study, found the boys: girls ratio of 1:6 in migraine, but only 1:3 in episodic tension-type headache (37). We reported that migraine was more prevalent in girls compared to boys by 2.1 times which is comparable to previous studies (38). A previous study found an increased risk of headache in females who had experienced menarche during the previous 2 years vs. females yet to reach menarche (29). We reported no significant difference between genders regarding prevalence of TTH. The prevalence of TTH in males and females varies between studies. Some studies reported a higher prevalence in females (38) while others reported no difference (29, 39). A population study in India reported that overall, headaches were found to be more common among girls, but tension-type was more common in boys (18).

We found a relation between gender and age, with the older girls experiencing headache more significantly than the boys, but no significant difference in gender among young children. Previous results are in accordance with our results that reported that with increasing age, the difference between boys and girls in the occurrence of headache increases (7, 29). The onset of puberty could be a triggering factor headache occurrence. We did not examine the relationship between the onset of menarche and headache occurrence in the present study, but higher prevalence of migraine headache and onset of menarche is clear. Headache is prevalent at preschool age, but entering puberty onset represents an increased risk of headache without great variation throughout puberty and in young adulthood.

Medical care utilization was reported in 67% of our cohort. A general practitioner was seen by 40% of the subjects. Similar result was reported in an Albers study, where 60% was seen by a physician (40) and in a German study, recurrent headache patients had consulted at least one physician in 57.7% of patients. Korean study reported an increasing number of patients visiting the hospital with headaches (22).

The majority of our cohort used symptomatic medications for headache, which is in agreement with earlier studies, that showed medication use is reported in a variable percentage going from 30 to 80% (41, 42). In a German study, 83.6% of the children used analgesics or anti-migraine drugs (29).

Headache may result in significant disability, including missed school days, and extra-curricular activities, suboptimal participation in regular activities, and loss of productivity (22). Previous population-based studies reported that students missed on average 7.2 school days in the last 6 months due to migraine, which is comparable to our study where the mean was 1.3 days in the last month (40).

The burden of headache in children and adolescents in our results is in agreement with earlier results that revealed total scores of 17.8 to 44 days where pediatric headache patients were totally or partially disabled at home or at school because of their headache (43).

## Strengths and Limitations and of the Study

Strengths of our research is that it is the first of its kind, since this is a population study investigating the prevalence of primary headache disorders in children and adolescents in Kuwait and it is highly representative of the Kuwaiti children and adolescent population, as it included all the six governorates of Kuwait. It is one of the first studies in the region that assessed the incidence rate and prevalence of pediatric primary headache disorders in a population-based setting. Information of increasing prevalence of pediatric primary headache disorders and burden may help in optimal planning for better management of patients in Kuwait and other countries in the region. The limitation that needs to be outlined is the sampling methodology. Although we propose a probability sample method, cluster sampling, to minimize this potential source of bias, we used a larger sample. Another limitation was the recall bias, which is usual in most of the studies using the questionnaire. Participation rate is <50% because our community is closed and not all subjects accepted to participate, to complete or give coherent data.

## Conclusions

Primary headache is common in children and adolescents in Kuwait. Primary headache prevalence increased with age in adolescent compared to children. Migraine is the most prevalent headache type. Primary headache prevalence showed no significant gender difference in children, however it was significantly higher in female adolescents. Primary headache can affect schoolwork, social activities and the parents' work. Awareness among general practitioners, pediatricians, families, and school teachers is crucial, since it can help to recognize headache in children and adolescents in its early stages and refer them for appropriate treatment to minimize the headache burden.

## Data Availability

Data are available on request from the Department of Neurology, Ibn Sina Hospital, Safat, Kuwait. P.O.Box 25427, 13115 Safat, Kuwait City, Kuwait.

## Ethics Statement

This study was carried out in accordance with the ethical guidelines of Kuwait University and Kuwait Ministry of Health. The protocol was approved by the ethics committee of Kuwait University. All subjects gave written informed consent in accordance with the Declaration of Helsinki.

## Author Contributions

JA-H designed the research and drafted the manuscript. SA contributed to research design, data acquisition, statistical analysis, and drafted the manuscript. RA drafted and revised the manuscript critically. All authors revised and approved the final manuscript.

### Conflict of Interest Statement

RA received honoraria as a speaker and for serving in scientific advisory boards from Bayer, Biogen, Biologix, Genzyme, Genpharm, Novartis, GSK, Merck-Serono. The remaining authors declare that the research was conducted in the absence of any commercial or financial relationships that could be construed as a potential conflict of interest.

## Abbreviations

CIs: Confidence intervals
GPs: General practitioners
HARDSHIP: Headache-Attributed Restriction, Disability, Social Handicap and Impaired Participation questionnaire; Mean
SDs: Standard Deviations
TTH: Tension Type Headache.
